# Supplementary material for: Ultra-compact dual-band smart NEMS magnetoelectric antennas for simultaneous wireless energy harvesting and magnetic field sensing
Source: Nat Commun. 2021 May 25;12:3141. doi: 10.1038/s41467-021-23256-z (PMC8149822; doi:10.1038/s41467-021-23256-z)
Supplement: Supplementary file 1 — Supplementary Information [file 41467_2021_23256_MOESM1_ESM.pdf]

Appendixes and Supplementary Materials:

A- Simulation Results of Strain and Displacement Distribution in FeGaB and AlN Thin-films

The results in Fig. S1 show the Multiphysics COMSOL simulation results (using coupled ACDC, Solid Mechanics, and Electrostatic modules) of strain and displacement distribution in FeGaB and AlN thin-films during energy harvesting process, where a 60nT magnetic field at 2.51GHz

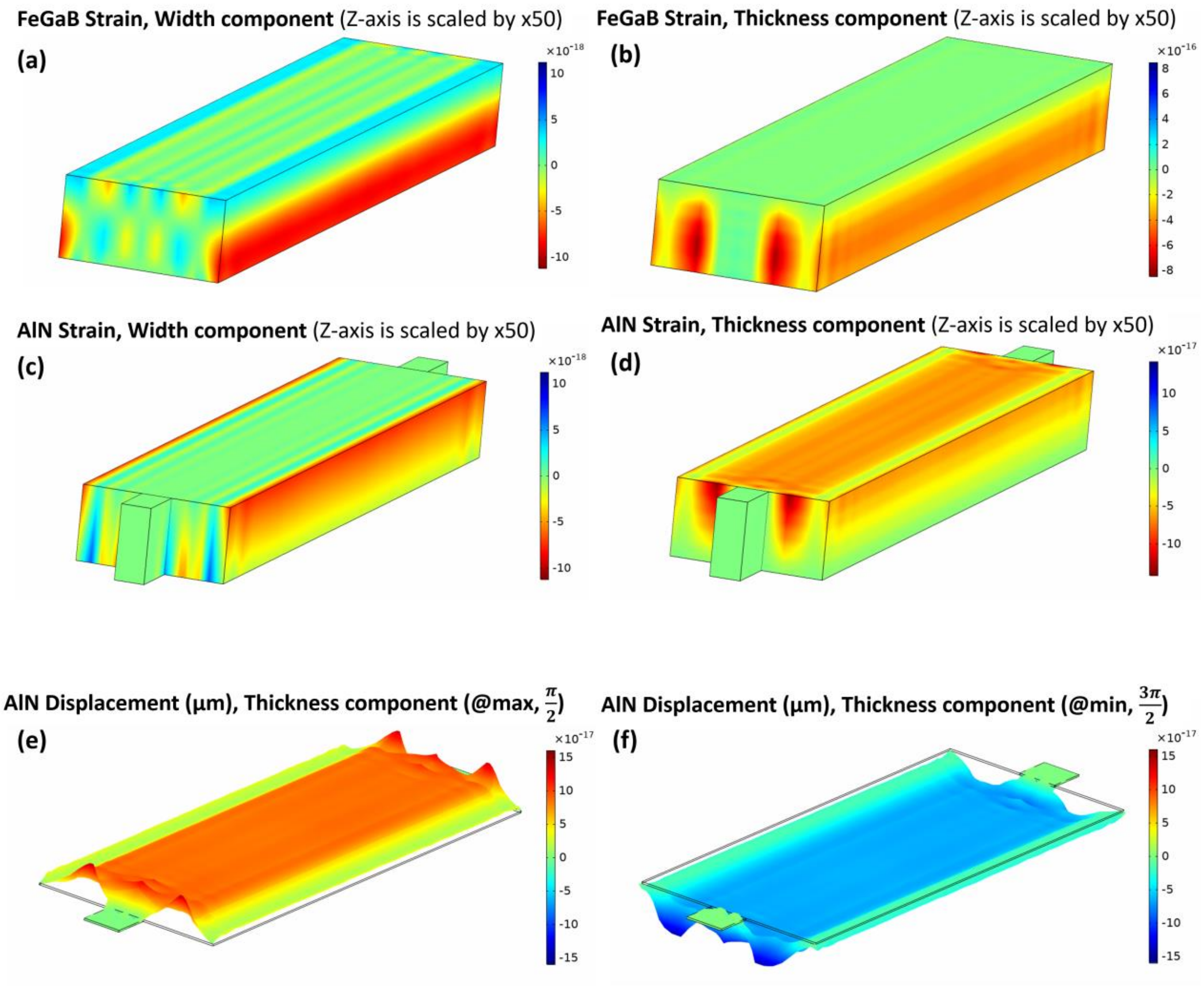

Fig. S1. Strain and displacement distribution in FeGaB and AlN thin-films. (a) and (b) show the induced strain along the width and thickness of FeGaB layer, respectively; (c) and (d) show the induced strain along the width and thickness of AlN layer, respectively. The induced strain along the thickness mode of AlN thin-film generates a voltage at 2.51GHz frequency which can be used for energy harvesting applications. (e) and (f) show the displacement along thickness direction of AlN thin-film at $\frac{\pi}{2}$ and $\frac{3\pi}{2}$ phase where displacement is maximum and minimum, respectively.

frequency is applied along the width of ME antenna. As the data in Fig. S1a and S1b show, this magnetic field creates a strain along the width and thickness directions of FeGaB material. Since the thickness of thin-films is significantly smaller compared to the width and length, the z-axis is scaled by x50 for a better visualization of strain distribution along the thickness. The induced strain in FeGaB layer is then transferred to the AlN thin-film, which is shown in Fig. S1c and S1d. The strain along the thickness mode of AlN induces a voltage at 2.51GHz frequency which can be used for energy harvesting purposes. Fig. S1e and S1f show the displacement along thickness direction of AlN thin-film at $\frac{\pi}{2}$ and $\frac{3\pi}{2}$ phases where displacement is maximum and minimum, respectively. The time domain animation of strain and displacement distribution over one full-cycle is also available in the supplementary materials as gif files.

B- Fabrication of a Smart CMR/FBAR ME Antenna

The fabrication process flow for the smart CMR/FBAR ME antenna is visualized in Fig. S2. The process starts with a high resistivity Silicon (Si) wafer (>10000 Ohm-cm). A 50 nm thick Pt film was sputter-deposited and patterned by lift-off on top of the Si substrate to define the bottom electrodes. Then, the 500 nm AlN film was sputter-deposited and vias were etched with H_3PO_4 to access the bottom electrodes. Afterwards, the AlN film was etched by Inductively Coupled Plasma (ICP) etching using Cl_2 based chemistry to define the shape of the resonant nano-plate. Next, a 100 nm thick gold (Au) film was evaporated and patterned by lift-off to form the top ground. Finally, 500 nm thick FeGaB/Al_2O_3 multilayer layer was deposited by a magnetron sputtering and patterned by lift-off. A 100 Oe in-situ magnetic field bias was applied during the magnetic deposition along the width direction of the device to pre-orient the magnetic domains. Then, the structure was released by XeF_2 isotropic etching of the Silicon substrate. The silicon underneath the rectangular resonators is completely etched and there is no leftover. Fig. S2f shows the device layout of the ME FBAR antenna with the detailed dimensions.

The magnetic multilayer with the structure of [FeGaB (45 nm)/Al_2O_3 (5 nm)] ×10 was sputter-deposited on AlN thin film with a Ta (5nm) seed layer at the Ar atmosphere of 3 mTorr with a background pressure of $<1\times10^{-7}$ Torr. The Ta seed layer promoted the FeGaB thin film growth exhibiting narrow resonance linewidth and close-to-bulk magnetic moment. The FeGaB layer was

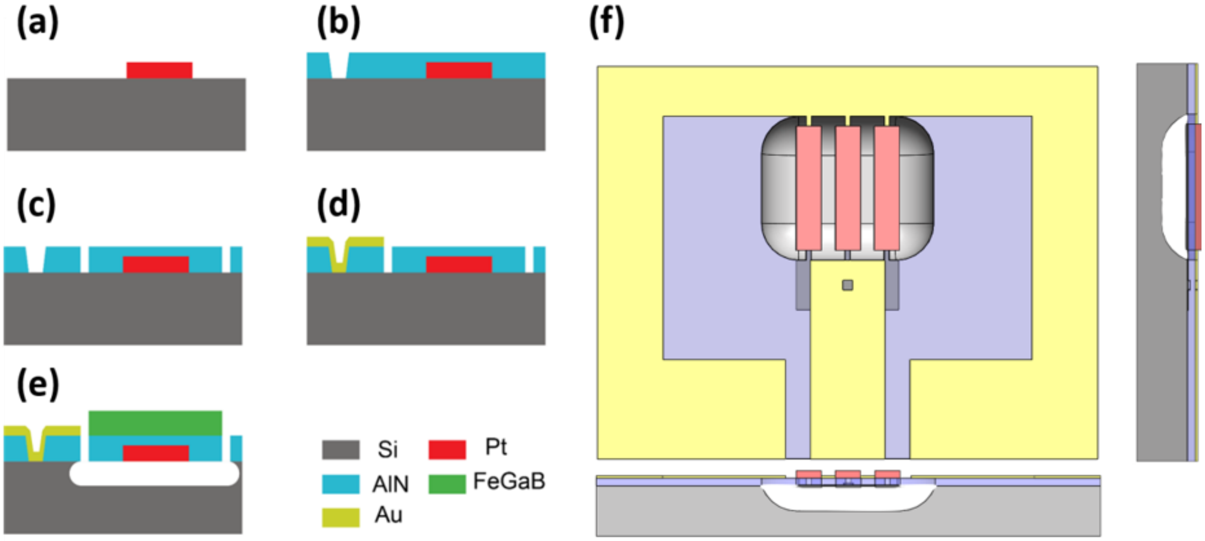

Fig. S2. Smart CMR/FBAR ME antenna fabrication process: (a) Pt thin film deposition and patterning. (b) AlN thin film deposition and wet etch of vias. (c) AlN thin film ICP etching. (d) Top electrode Au deposition and patterning. (e) Magnetic multilayer FeGaB/AlO_x deposition and pattering followed by Si substrate release. (f) Device layout of the ME FBAR antenna.

co-sputtered from FeGa (DC sputtering) and B (RF sputtering) targets. The Al_2O_3 layer was deposited by RF sputtering using an Al_2O_3 target. The deposition rates are calibrated by X-ray reflectivity.

The reason we have used FeGaB/Al2O3 multilayers (discussed in Appendix B, fabrication of ME antenna) is that they demonstrate eddy-current loss reduction, lower out of plane anisotropy, and enhance permeability in comparison with a single FeGaB layer with the same thickness. Therefore, we are not worried about the eddy-current loss. We have been extensively researching ME antennas and ME sensors optimization since 2015, and we are one of the leading teams on this effort. As for the electrode, we have tried three different approaches: (1) Using FeGaB film as an electrode; (2) Using gold between FeGaB and AlN for better conductivity; (3) Using gold as an electrode on top of the FeGaB for better conductivity. The results always showed better performance, sensitivity, and quality factors using FeGaB directly as the electrode. Therefore, in this project, we used FeGaB directly instead of using other metal on top or in-between.

C- Misalignment and Rotation of the ME Antenna in other Directions (for Energy Harvesting Tests)

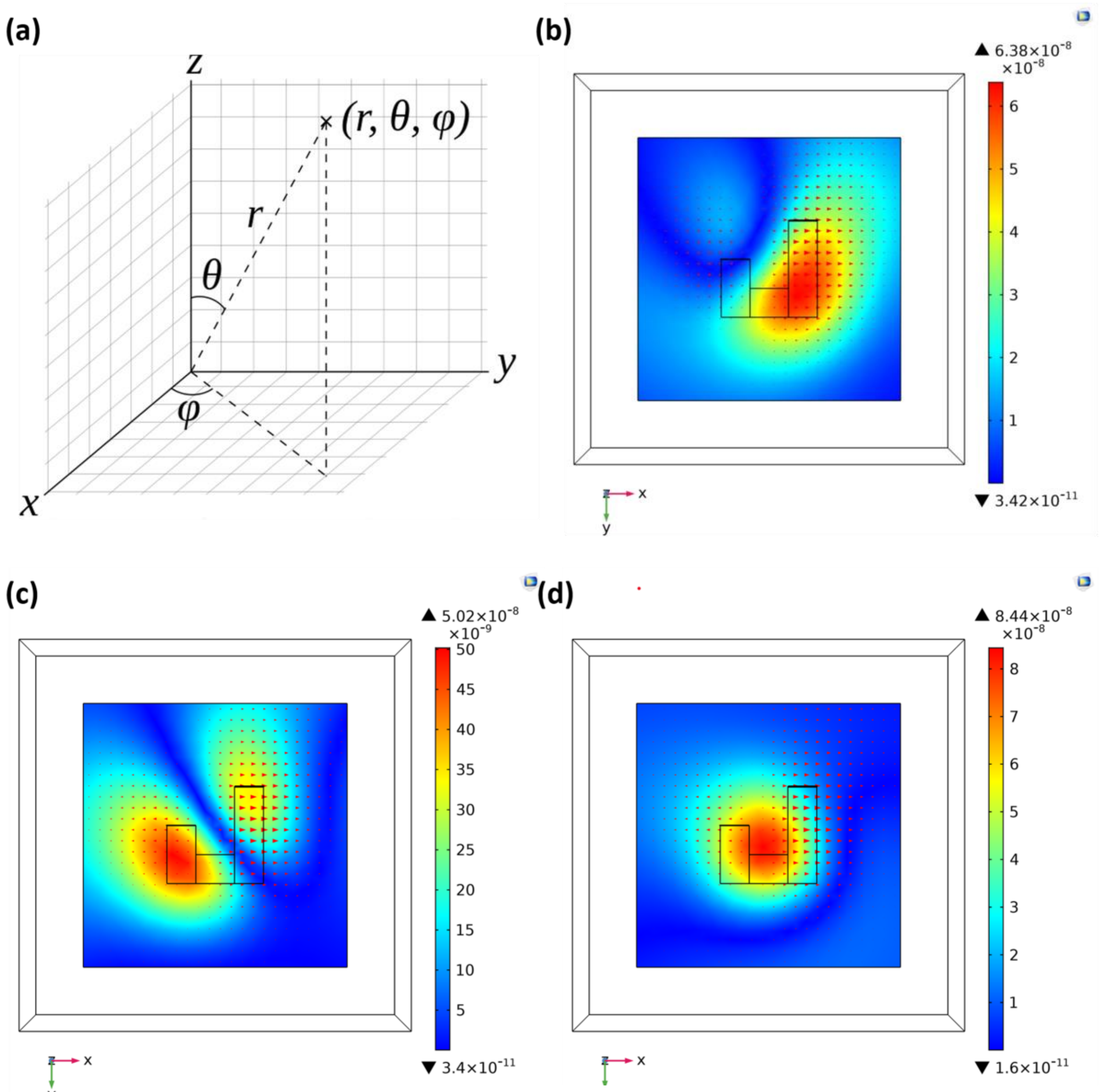

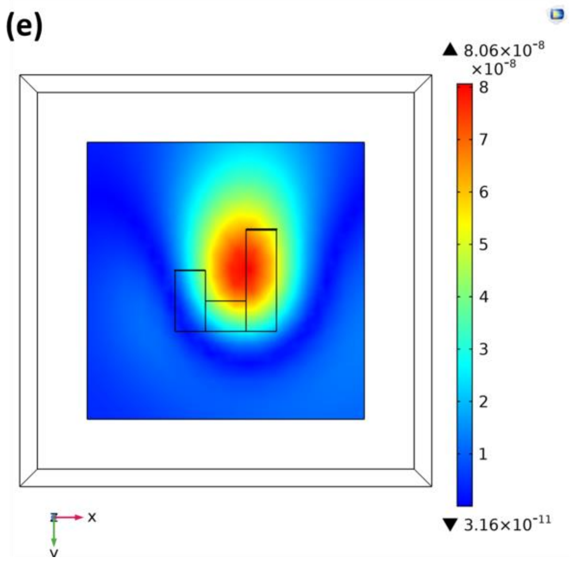

Fig. S3. (a) The spherical coordinate systems used for demonstrating different orientations, where the vector $\bar{r}$ shows the direction towards which the ME antenna is sensitive. ϕ and θ show the angles between $\bar{r}$ vector and x and z axis, respectively; (b) the orientation in which $\phi = 45^o$ and $\theta = 90^o$; (c) the orientation in which $\phi = 135^o$ and $\theta = 90^o$; (d) the orientation in which $\phi = 90^o$ and $\theta = 45^o$; (e) the orientation in which $\phi = 0$ and $\theta = 45^o$.

The magnetic flux density components (Bx, By, Bz) generated by Tx coil in the XY plane were shown and discussed in Section 2. In this appendix, we will discuss the magnetic flux density distribution in several other orientations. Fig. S3a shows the spherical coordinate system used for demonstrating different orientations, where the vector $\bar{r}$ shows the direction towards which ME antenna is sensitive. ϕ and θ are the angles between $\bar{r}$ vector and the x and z axes, respectively. Fig. S3b shows the orientation in which $\phi = 45^o$ and $\theta = 90^o$, i.e. this is the case where the ME antenna is 45° between the x- and y-axis. This orientation was discussed with more in details in Section 2. Fig. S3c shows the orientation in which $\phi = 135^o$ and $\theta = 90^o$. Fig. S3d shows the orientation in which $\phi = 90^o$ and $\theta = 45^o$. Fig. S3e shows the orientation in which $\phi = 0$ and $\theta = 45^o$.

D- Magnetic Field and Eddy Current Distribution Inside the Tissue and Air Mediums

As discussed in Section 2 of the article, the magnetic field distribution is different in air and tissue mediums. According to simulation results, eddy current loops generated inside the tissue impact the magnetic field distribution. These eddy current loops are generated by the initial magnetic flux density generated in the Tx coil, which are flowing towards the z-axis. Fig. S4a and S4b show the magnetic flux density on two different planes of air and tissue mediums, respectively. The vertical planes are the same cross-section planes shown in Fig. S4c and S4d. They show that the magnetic field distribution is normal in air but distorted in tissue medium. There are two vortexes in tissue medium, which are due to eddy current loops created in the meninges layer because of its higher conductivity. The horizontal planes show the magnetic flux density above the coil at the interface of meninges and grey matter layers, where the mentioned vortexes are created. Fig. S4c and S4d, horizontal planes, show the current density ($\frac{d\vec{D}}{dt} + J_c$, the first and second terms are displacement current density and conduction current density, respectively) on the interface of meninges and grey matter layers in air and tissue medium, respectively. The magnetic flux Bz, shown in Fig. S4b, creates eddy current loops rotating around the B-fields in the tissue. These eddy current loops in turn generate the magnetic field loops on the vertical plane which lead to the vortexes shown with black-loops. There are no generated eddy current loops in the air, and therefore the Tx coil field distribution is normal and not distorted.

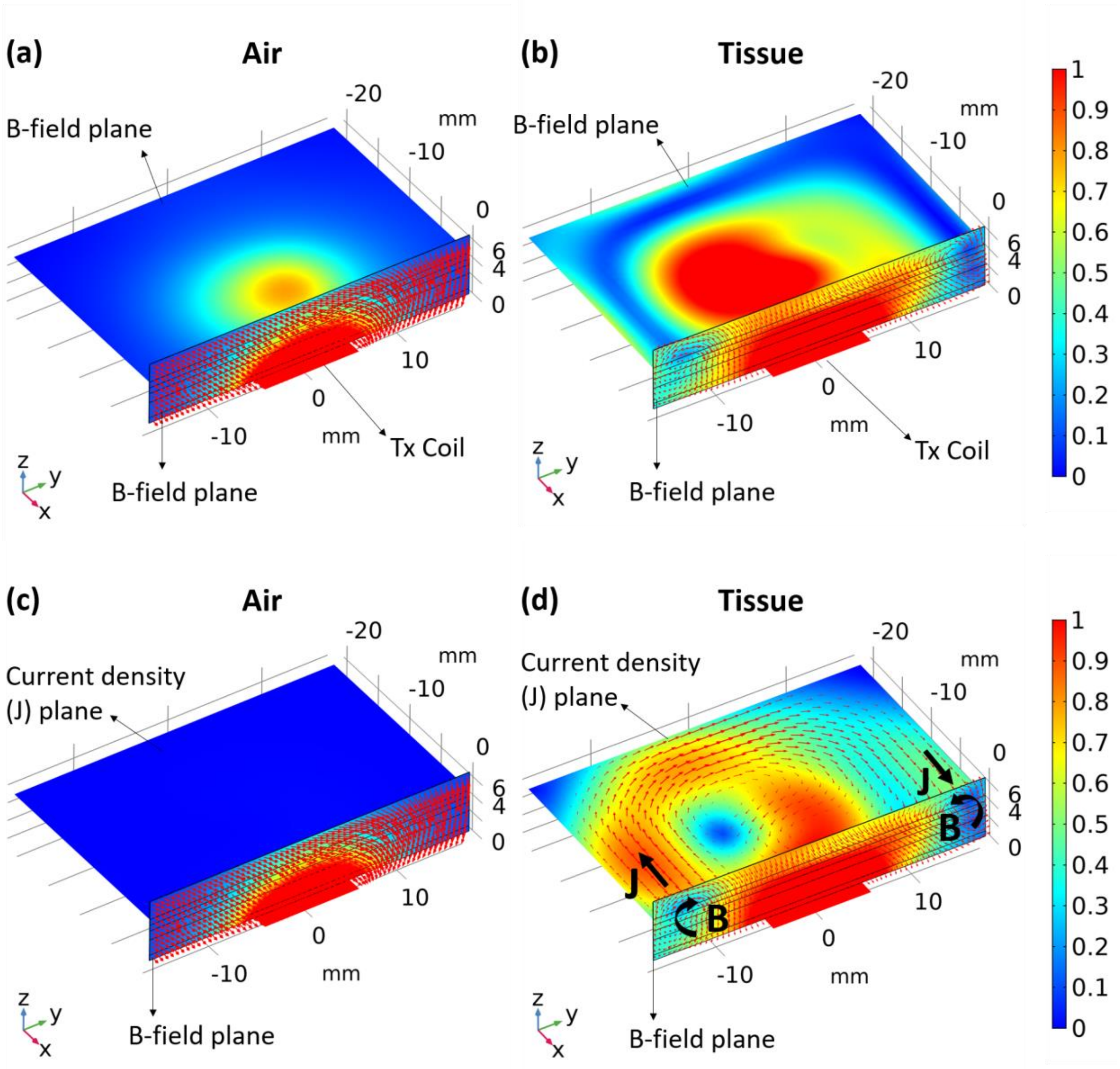

Fig. S4. (a) and (b) magnetic flux distribution on two vertical and horizontal cross-section planes in air and tissue mediums, respectively. The fields on horizontal planes are mostly towards z-axis; (c) and (d) show the current density on horizontal plane and magnetic flux density on vertical plane in the air and tissue mediums, respectively. The horizontal plane is the interface of meninges and grey matter layers. As it is shown the magnetic field Bz create eddy current loops on the horizontal plane in fig S3d. These eddy currents in turn generate magnetic field loops which interfere with the magnetic fields from Tx coil. Therefore, they distort the initial field distribution and create the two vortexes shown in the vertical plane.

E- Energy Harvesting and Magnetic Field Sensing Experimental Setup

The experimental setup for the energy harvesting measurements is displayed in Fig. S5. The Tx coil and ME antenna are mounted on two 3D printed plastic manipulators in order to precisely adjust their position. Plastic manipulator, instead of metallic ones, are used to minimize the effect of objects surrounding the Tx coil and their impact on S_{11} of the coil. The vector network analyzer (VNA) Agilent Technologies E8364A is used as an RF source in this experiement to both provide power to the Tx coil and track the S11 of the coil in real-time to ensure proper impedance matching during the experiment.

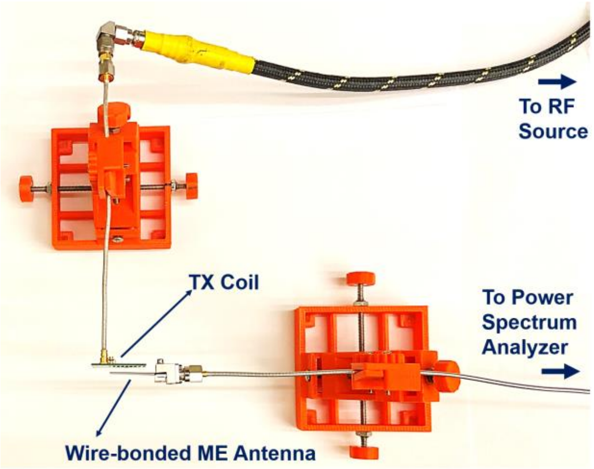

Figure S5. Experimental setup used for the energy harvesting measurements.

The diagram of experimental setup for magnetic sensing using ME antenna is shown in Fig. S6. The zoomed-in part on the right shows the device under test and its orientation with respect to the

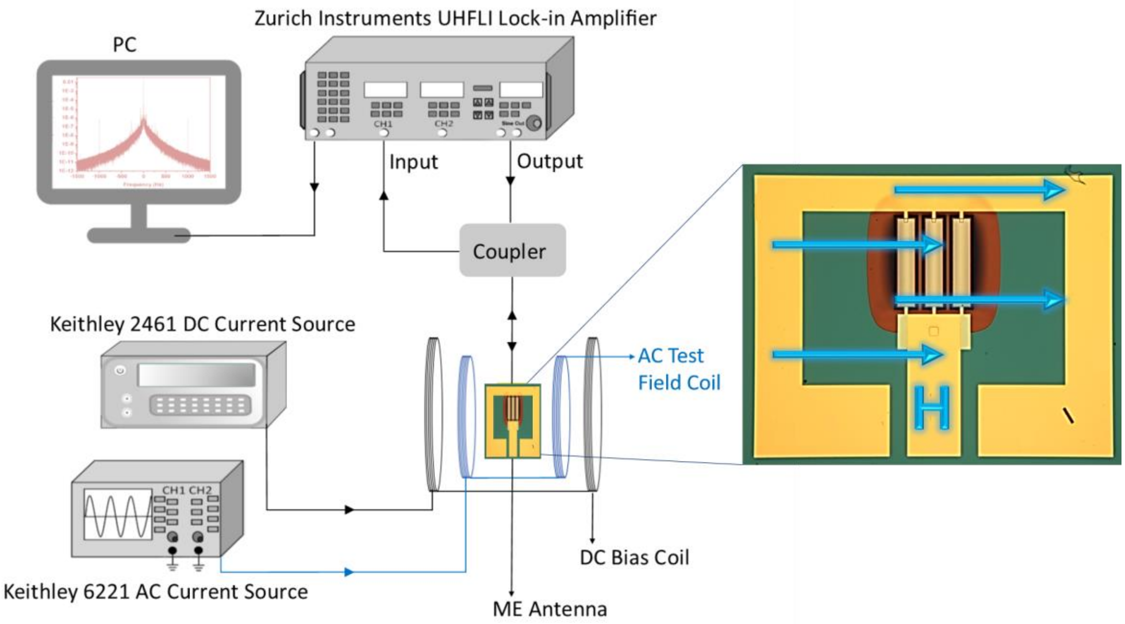

Figure S6. Experimental setup used for magnetic sensing using an ME antenna, where the zoomed-in part on the right shows the device under test and its orientation with respect to external magnetic field. It is notable that this is the same direction as during the energy harvesting tests.

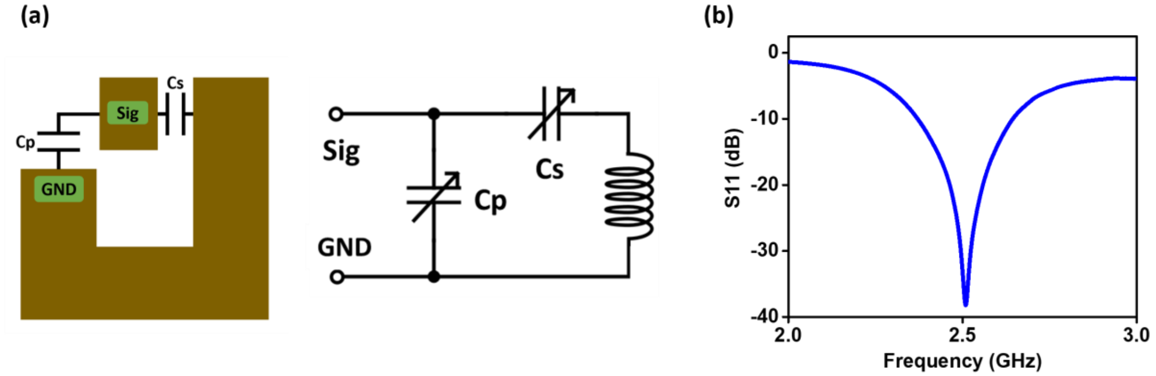

Figure S7. (a) Diagram of the PCB traces of the Tx coil used in the energy harvesting experiment, and schematic of the L-matching network for impedance matching of the coil; (b) measured S11 of the Tx coil matched at 2.51 GHz, which is the FBAR resonance frequency of the smart ME antenna.

external magnetic field. The part number of the coupler used in the experiment is ZDC-10-1+ from Mini-Circuits. A 37 Oe DC bias field provided by the larger Helmholtz coil (black color) is applied perpendicular to the length of the resonator in order to maximize the magnetoelectric coefficient and sensitivity. The alternating magnetic field H_m, which is the external test magnetic field, is in the same direction as DC bias field and is provided by smaller Helmholtz coil (blue color). The larger and smaller Helmholtz coils are driven by a Keithley 2461 DC current source and Keithley 6221 AC current source, respectively.

F- Transmitter Coil Design

A single turn T_X coil on an FR4 PCB is designed to investigate the energy harvesting performance and efficiency of ME antennas. The Sonnet simulation toolbox was used to optimize the Tx coil in terms of size, Q-factor, self-resonance frequency (SRF), and trace width. The optimized and fabricated Tx coil, shown in Fig. S7a, has a self-resonance frequency (SRF) of 4.1 GHz, Q-factor and inductance of 110 and 10.49 nH (both @ 2.51 GHz and in air), length of 10 mm, and trace width of 3 mm. An L-match capacitive network, also displayed in Fig. S7a, is used to match the Tx coil at 2.51 GHz for maximization of the transmitted power. An SMP connector is soldered to the PCB to connect the Tx coil to the VNA for impedance matching and characterization. Fig. S7b shows the reflection coefficient (S_{11}) of the T_X coil matched at 2.51GHz, which is the FBAR resonance frequency of the smart ME antenna as seen in Fig. 2b. Note that S11 in Fig. S7b was measured when the Tx coil was surrounded by air. When the Tx coil is placed close to the skin or tissue, the resonance frequency of the matched Tx coil would shift by 100-300 MHz compared to the air medium; therefore, one has to re-match the Tx coil when tissue is present. For this reason,

the matching network in Fig. S7a has been implemented with variable capacitors. As mentioned in the manuscript, the GSG pads of a smart CMR/FBAR ME antenna, shown in Fig. 1b, are wire-bonded to a PCB, and an SMA connector is mounted on the PCB to obtain access to ME antenna's pads.

G- Electromagnetic FEM Simulations of Brain Tissue Layers

Electromagnetic simulations of brain tissue layers were performed using COMSOL Multiphysics v5.4 software under AC/DC module [Magnetic and Electric fields (mef) physics]. A 10 mA at 2.51 GHz was applied to the Tx coil and the Maxwell equations were solved in the air and tissue layers. The electric and magnetic field distributions in the tissue are within the reactive region given the small distances from the Tx coil, and not in the radiation region. The electromagnetic properties (At 2.51 GHz) and thickness of the brain layers are shown in Table S1. The electromagnetic properties of the brain layers used in the simulation are extracted from the IT'IS Research Foundation Dataset [DOI: 10.13099/VIP21000-04-0. itis.swiss/database].

Table 1. The thickness and the electromagnetic properties (at 2.51 GHz) of the brain layers.

Layer	Thickness (mm)	Relative Permittivity	Conductivity (S/m)
Scalp	1.7	37.9	1.49
Skull	1	11.3	0.406
Dura Mater	0.65	42	1.7
CSF	0.65	66.2	3.52
Grey Mater	3	48.8	1.85

H- Experimental Setup for the Simultaneous Energy Harvesting and Magnetic Field Sensing

The diagram of experimental setup for simultaneous energy harvesting and magnetic field sensing experiment is shown in Fig. S8. In this experiment, we have merged the experimental setups explained in Appendix E by adding a splitter to the circuit. The rest of the setup is similar to the

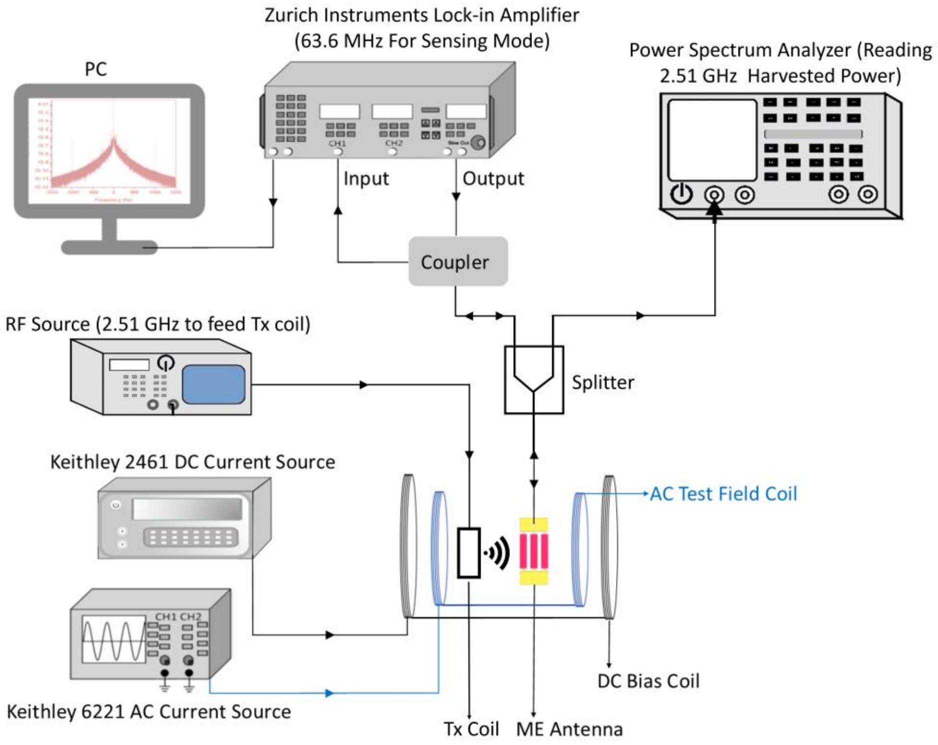

Figure S8. Experimental setup used for the simultaneous energy harvesting and magnetic field sensing experiment. In this setup we have merged both experimental diagrams shown in Appendix E via a splitter.

diagrams shown in Appendix E. In the simultaneous experiment, the RF signal going out of the ME antenna includes both 2.51 GHz and 63.3 MHz frequencies where the former component is produced by the thickness mode of ME antenna via wireless energy harvesting and the latter one is the reflected component of width mode resonance frequency which includes the modulation peaks carrying the information of the external low frequency magnetic field. It is notable that, similar to the magnetic field sensing experiment shown in Appendix E, here the ME antenna's width mode is excited by the 63.6 MHz RF signal coming from the lock-in amplifier via splitter, reflection of which goes back to the input of the lock-in amplifier and carries the modulation components.

I- Proposed Integrated Circuit (IC) Design for Wireless Data Communication and Energy Harvesting

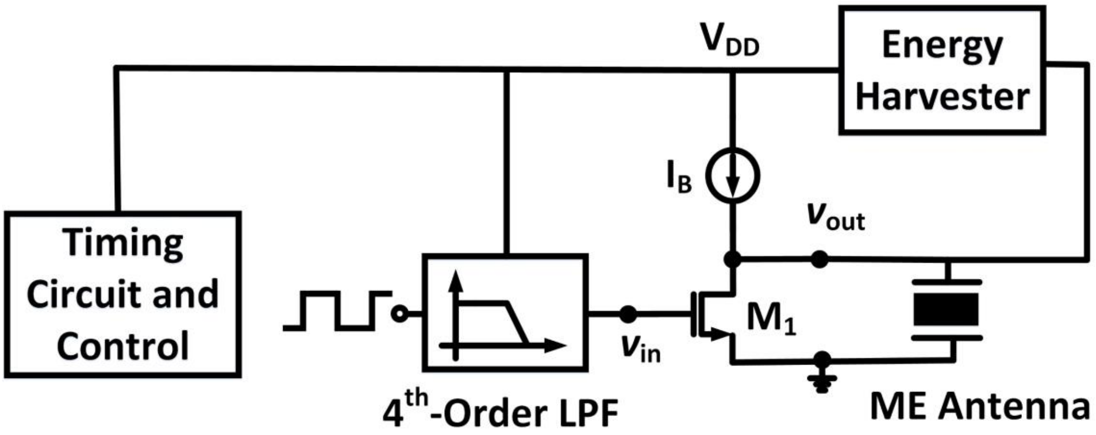

Figure S9. Conceptual diagram of an ME antenna integrated with a chip for magnetic field sensing and wireless energy harvesting.

We have explored data transmission via a circuit design technique. The proposed method is to synthesize a local oscillator (LO) using the ME antenna operating at its contour mode resonator (CMR) resonance frequency, and then to use this LO to apply a sinusoidal-like excitation current at the ME antenna. This produces an amplitude-modulated output when there is a change in the magnetic field due to neuronal activity. As visualized in Fig. S9, the device with the ME antenna will require circuitry for energy harvesting at the FBAR resonance frequency, as well as a driver circuit that generates a carrier signal at the CMR resonance frequency for transmission of the sensed information. The magnetic modulation-based capability to transmit sensed data from the ME antenna with the described approach has been assessed by simulating an ME antenna model with the driver transistor stage (M_1), for which our results are published in [28].

With the above-mentioned transmission of the sensed information, the required external receiver can perform standard operations for processing the amplitude-modulated signal generated in [28]. The magnetically amplitude-modulated received signal has to be amplified and down-converted by the external RF receiver front-end, and further processed with digital filtering to extract the signal components that carries the sensed magnetic field information. For the first implementation of these operations, we suggest the use of Universal Software Radio Peripheral (USRP) for software-defined radio processing of the amplitude-modulated signal.

Using the proposed IC design we can also align the external Tx coil to the implanted device in order to get the highest wireless power transfer efficiency. The device generates the largest power

when the ME antenna is in the most optimum angle for the energy harvesting, which in turn it transmits back the strongest amplitude-modulated signal. In other word, based on the strength of the externally received amplitude-modulated signal we can align the Tx coil to the implanted device to achieve the highest power transfer efficiency.
